# Transmissibility of caprine scrapie in ovine transgenic mice

**DOI:** 10.1186/1746-6148-8-42

**Published:** 2012-04-02

**Authors:** Katherine I O’Rourke, David A Schneider, Terry R Spraker, Rohana P Dassanayake, Margaret A Highland, Dongyue Zhuang, Thomas C Truscott

**Affiliations:** 1 United States Department of Agriculture, Agricultural Research Service, Pullman, WA, 99164, USA; 2 Department of Microbiology, Immunology and Pathology, College of Veterinary Medicine and Biomedical Sciences, Colorado State University, Fort Collins, CO, 80526, USA; 3 Department of Veterinary Microbiology and Pathology, College of Veterinary Medicine, Washington State University, Pullman, WA, 99164, USA

**Keywords:** Prion, Mouse, Transgenic, Caprine

## Abstract

**Background:**

The United States control program for classical ovine scrapie is based in part on the finding that infection is typically spread through exposure to shed placentas from infected ewes. Transmission from goats to sheep is less well described. A suitable rodent model for examining the effect of caprine scrapie isolates in the ovine host will be useful in the ovine scrapie eradication effort. In this study, we describe the incubation time, brain lesion profile, glycoform pattern and PrP^Sc^ distribution patterns in a well characterized transgenic mouse line (Tg338) expressing the ovine VRQ prion allele, following inoculation with brain from scrapie infected goats.

**Results:**

First passage incubation times of caprine tissue in Tg338 ovinized mice varied widely but second passage intervals were shorter and consistent. Vacuolation profiles, glycoform patterns and paraffin-embedded tissue blots from terminally ill second passage mice derived from sheep or goat inocula were similar. Proteinase K digestion products of murine tissue were slightly smaller than the original ruminant inocula, a finding consistent with passage of several ovine strains in previous reports.

**Conclusions:**

These findings demonstrate that Tg338 mice propagate prions of caprine origin and provide a suitable baseline for examination of samples identified in the expanded US caprine scrapie surveillance program.

## Background

Ovine and caprine scrapie are transmissible spongiform encephalopathies (TSEs) of small ruminant livestock. TSEs are also reported in domestic cattle and exotic zoo-raised bovids (bovine spongiform encephalopathy or BSE) and in farm-raised and free-ranging cervids (chronic wasting disease or CWD of deer, elk and moose). Human TSEs include a varied group of familial, sporadic, iatrogenic and infectious disorders. Human variant Creutzfeldt Jakob disease (vCJD) is a novel infectious TSE apparently originating from exposure to infectious bovine tissues [1,2] and transmitted at low rates through blood transfusion [3]. Although scrapie has not demonstrated zoonotic potential, the introduction of vCJD through exposure to domestic animal food products has led to a call for global eradication of all animal TSEs.

TSEs are characterized by accumulation of a relatively protease resistant isoform (PrP^Sc^) of the normal cellular prion protein (PrP^c^), encoded by the PRNP gene [4]. The transmissible agent, referred to as a prion, is uniquely proteinaceous [5] and composed largely of PrP^Sc^. In all TSEs, prions and PrP^Sc^ accumulate in the central nervous system but significant accumulation also occurs in the lymphoid tissue and placenta [6,7] of sheep and goats with classical scrapie. Classical ovine scrapie is apparently transmitted by oral or mucosal exposure to prions shed in the placenta, blood and/or milk of infected postparturient ewes [8]. Susceptibility to infection with the classical ovine scrapie agent is associated with the amino acid sequence of the host PrP^c^[9]. Selection for relatively resistant breeding stock has the potential of accelerating eradication of ovine scrapie from domestic flocks.

Caprine scrapie may originate from exposure to infected sheep or infected goats. Classical ovine scrapie is readily transmitted from sheep to domestic goats by experimental oral challenge with placental tissue [8]. The practice of co-housing sheep and goats during parturition and the use of milk from goats for feeding orphaned lambs raise the question of whether caprine scrapie is transmissible to sheep under field conditions and if so, whether there are strains of caprine scrapie that result in novel diagnostic or transmission patterns when passaged in sheep. Large animal experiments to address these issues in the natural host are expensive and time consuming, with incubation time measured in years. The introduction of transgenic mice in which the murine PRNP gene is replaced by the PRNP gene of the species of interest has provided an alternative means for estimating interspecies transmission potential. The purpose of this study was to examine the feasibility of using the well characterized ovinized mouse strain Tg338 as a surrogate for sheep as bioassay recipients for detection of caprine prions.

## Results

### Donor animals

Brain tissue was collected from three domestic goats (case numbers 3538, 3558 and 30–75) and one reference domestic sheep (case number 3178) with naturally-acquired classical scrapie. All four animals had been identified as scrapie suspect animals based on antemortem detection of PrP^Sc^ in rectal mucosal associated lymphoid tissue using immunohistochemistry. Scrapie was diagnosed by postmortem detection of PrP^Sc^ in the medulla at the level of the obex.

PRNP genotyping was performed on the 4 donor samples. The reference sheep 3178 was homozygous for the wild type ovine allele, encoding alanine at codon 136, arginine at codon 154, and glutamine at codon 171 (ARQ/ARQ). This genotype is identified in more than 90% of the scrapie cases diagnosed in the US [10] and Canada [11]. The caprine PRNP gene is highly polymorphic [12,13] and there is no standard nomenclature for naming alleles. Polymorphisms have been identified on two haplotypes, differing at codon 240 (encoding proline P or serine S). Goat 3538 was homozygous for the wild type allele encoding 240P with no other polymorphisms on either strand. Goat 30–75 was heterozygous for 2 alleles on the 240P backbone; the wild type allele encoding glycine at codon 127 and an alternative allele encoding serine at that site. Goat 3558 was heterozygous for 2 alleles on the 240P backbone; the wild type allele encoding isoleucine at codon 142 and an alternative allele encoding methionine at that site.

**Figure 1:**
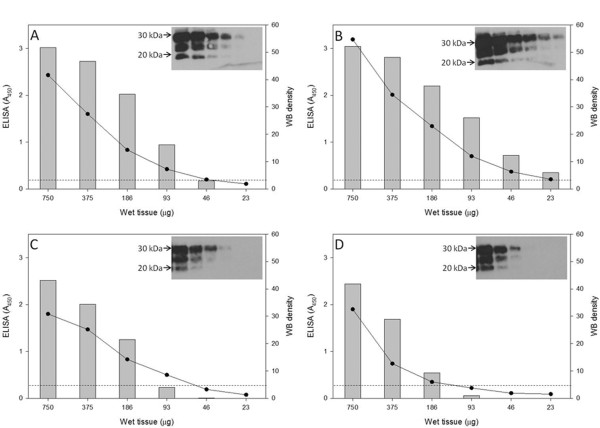
**Relative levels of PrP**^**Sc **^**in brain homogenate from donor animals.** Serial two-fold dilutions of brain homogenate from goat 3538 **(A)**, 3558 **(B)**, 30–75 **(C)** and reference sheep 3178 **(D)** were analyzed by western blot analysis and ELISA. Absorbance (A_450_) results from ELISA (−·-) are shown on the left y axis and density of the diglycosylated band determined from scans of western blot (vertical bars and inset image) on the right y axis. Starting wet weight equivalents per ELISA well or Western blot lane are shown on the x axis. Cut off limit for the ELISA (−−----------) was determined from the manufacturer’s negative control sample.

Brain homogenates were assessed for relative PrP^Sc^ levels using serial endpoint dilutions in western blot and ELISA (Figure 1, panels A-D). ELISA testing using a commercial kit was performed on serial two-fold dilutions of brain homogenate; the coefficient of variation (CV) for the 24 samples (6 dilutions per homogenate for each of 3 goats and 1 sheep) ranged from 0 to 0.19, with 20 of the 24 samples having CV of less than or equal to 0.1 (data not shown). The last serial dilution resulting in a detectable signal by either ELISA or western blot analysis varied from 23 (for goat 3558) to 186 (for goat 3538 and sheep 3178) μg starting wet weight tissue. Inoculations were performed using a standard brain homogenate inoculum equivalent to 2 mg starting wet weight containing 71–99 mg total protein (data not shown).

### Survival curves in bioassay mice

**Figure 2:**
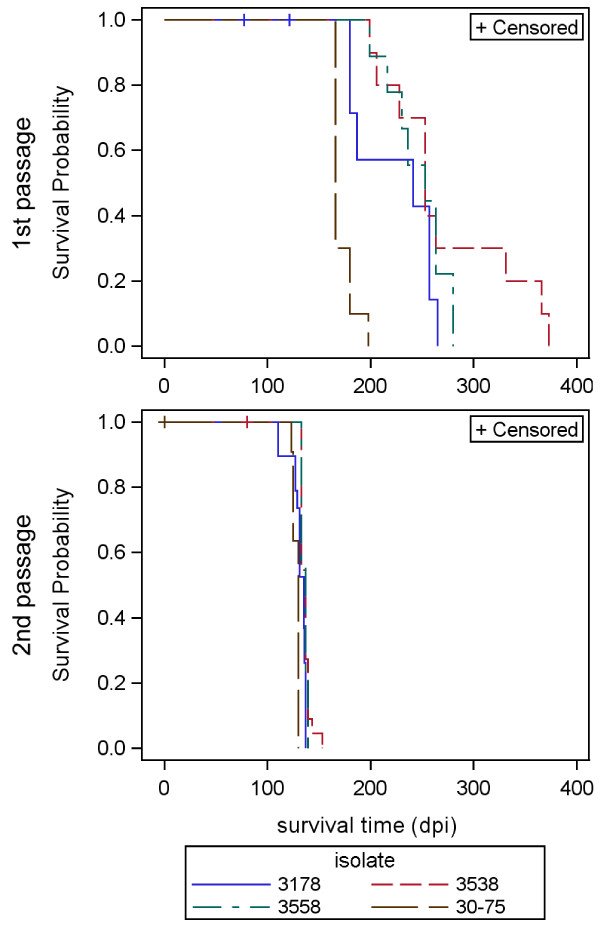
**Kaplan-Meier survival curves for Tg338 mice inoculated with different isolates of small ruminant scrapie.** Shown are the survival curves for first (upper panel) and second (lower panel) intracerebral passage in Tg338 mice including censored data. Scrapie isolates were derived from the brain of three clinical goats (broken lines: 3538, 3558 and 30–75) and a reference sheep (solid blue line: 3178). Note the reductions in survival times per isolate (days post-inoculation, dpi) as well as the reduction in survival time variation between isolates upon second passage. The color line code for each isolate source is indicated in the key.

Shown in Figure 2 are the first (P1) and second (P2) passage survival curves for Tg338 mice inoculated by the intracerebral route with scrapie isolates derived either from the three naturally infected goats (3538, 3558, 30–75) or a naturally infected reference sheep (3178). Median survival times with 95% confidence intervals are reported for all isolates in Table 1. P1 survival curves were relatively prolonged in duration and variable (median survival time: range, 166–253 dpi), with limited overlaps between curves produced by the different isolates. In contrast, P2 survival curves were considerably less variable and shorter in duration (median survival times: range, 130–137 dpi). Per isolate, the median survival time was reduced upon second passage by 36–116 dpi. Since the P1 survival curve for the isolate derived from goat 3538 appeared biphasic, the P1 brain homogenate from two mice, a shorter survival time mouse (228 dpi) and a longer survival time mouse (331 dpi), were used in P2 for this caprine scrapie sample. A significant difference was not observed between these two subset P2 survival curves (data not shown; Wilcoxon Chi-square = 2.1399, *P* = 0.1453). Thus, these two data subsets were combined for calculation of the median survival time reported for this sample in Table 1.

**Table 1:** Inoculum groups and median survival times after serial intracerebral passage in Tg338 mice

**Inoculum groups**		**Passage**	**Recipients tested**^**1**^	**Median survival time**	**95%** **LCL-UCL**^**2**^
**Donor** **species**	**Donor** **case number**		***(n, c)***	**(dpi)**	**(dpi)**
goat	30–75	P1	10, 0	166.0	166.0–180.0
	3538		10, 0	253.0	199.0–331.0
	3558		9, 0	253.0	199.0–280.0
sheep	3178		7, 3	241.0	180.0–257.0
goat	30–75	P2	11, 3	130.0	125.0–130.0
	3538		22, 2	137.0	133.0–137.0
	3558		9, 0	137.0	133.0–139.0
sheep	3178		19, 0	135.0	129.0–136.0

^1^ Kaplan-Meier product-limit estimation of survival functions for *n* non-censored and *c* censored recipients per isolate.

^2^95% lower and upper confidence limits (UCL, LCL) were determined using the log(−log) transform.

### Western blot analysis

**Figure 3:**
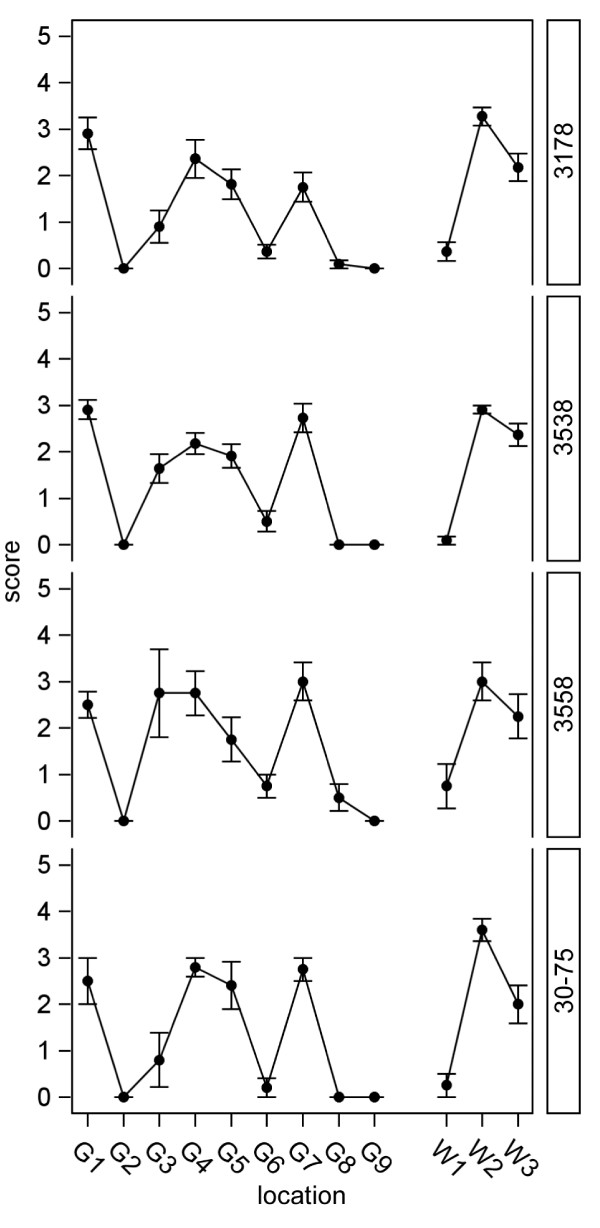
**Western blot analysis of brain from small ruminants and P2 Tg338 mice bioassay recipients.** Western blot analysis of brain from goat 3538 (lane 1, 150 μg starting wet weight brain ), goat 3558 (lane 3, 250 μg ), goat 30–75 (lane 5, 250 μg) and sheep 3178 (lane 7, 350 μg), and brain from P2 mice from each of those 4 inoculum groups (lanes 2, 4 , 6 and 8, respectively, loaded with 60–100 μg brain) after digestion with proteinase K (50 μg/ml final concentration for goats and sheep, 100 μg/ml final concentration for mice) and detection with mAb F99/97.6.1, binding a carboxyl epitope **(A)** or mAb P4, binding an amino terminal epitope **(B)**. Lane 9 contains brain (300 μg) from an age-matched uninoculated Tg338 mouse, homogenized and treated with proteinase K as described for tissue from P1 and P2 mice. Molecular mass markers are shown on the left.

Western blot analysis of brain homogenates from donor goats (n = 3) and sheep (n = 1) and bioassay recipient Tg338 mice (n = 17 P1, 21 P2 mice) showed the characteristic banding pattern of proteinase K resistant PrP^Sc^. Representative data are shown in Figure 3. Ovine and caprine brain samples co-migrated and were detected with antibodies binding the carboxyl terminus (mAb F99/97.6.1, residues 221–224) (Figure 3A), the amino terminus (mAb P4, residues 93–99) (Figure 3B), and the central region (SAF84, residues 167–172) (data not shown). For samples derived from all Tg338 mice, the unglycosylated band migrated with an apparent molecular weight approximately 1.5–2 kDa lower than the samples from sheep and goat donor tissues. Epitope mapping demonstrated the loss of the epitope for amino terminus mAb P4 (Figure 3B) but conservation of the epitope for mAb F99/97.6.1 (Figure 3A). There was no evidence of a 14 kDa carboxyl fragment when using mAb SAF84 [14]. 

**Figure 4:**
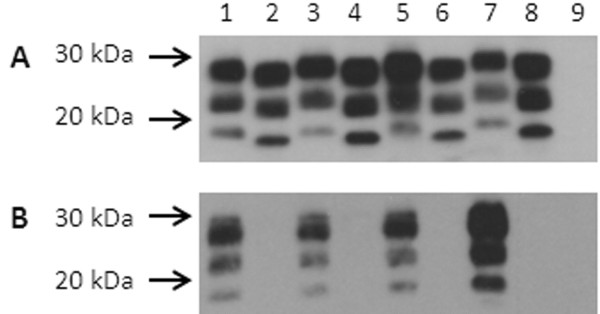
**Co-migration of brain homogenates from Tg338 mice inoculated by the intracerebral route or the intraperitoneal route.** Brain homogenate from sheep 3178 (lanes 1, 3, 5 and 7, 350 μg starting wet weight per lane) and Tg338 mice (100 μg starting wet weight per lane) inoculated by the intracerebral route and assayed at passage 2 (lane 2) or passage 1 (lane 4). The same homogenate was inoculated intraperitoneally in Tg338 mice; first passage brain (lane 6) and spleen (lane 8) homogenates were assayed. Lane 9, brain homogenate (200 μg starting wet weight) from uninoculated Tg338 mouse. All tissues were digested with proteinase K (100 μg /ml final concentration for murine tissues and 50 μg/ml final concentration for ovine tissues). Filter was probed with mAb F99/97.6.1. Molecular mass markers are shown on the left.

The possibility that the apparent shift in the PK cleavage site on mouse passage was due to replication in peripheral tissues and subsequent dissemination to the brain was addressed by intraperitoneal challenge of mice with the reference sheep homogenate. Electrophoretic profiles of PrP^Sc^ from the brain tissue of P1 (Figure 4, lane 4) or P2 (Figure 4, lane 2) mice inoculated with the reference sheep brain homogenate (Figure 4, lanes 1, 3, 5 and 7) by the IC route (Figure 4, lanes 2 and 4) were similar to those from the brain of P1 mice inoculated by the intraperitoneal (IP) route (Figure 4, lane 6), demonstrating co-migration of brain-derived PrP^Sc^ from murine samples regardless of route of inoculation. PrP^Sc^ bands from Tg338 spleen samples (Figure 4, lane 8) had a slightly slower migration rate than those from brain samples, ruling out peripheral amplification as a mechanism for the profile shift.

### Glycoform analysis

**Figure 5:**
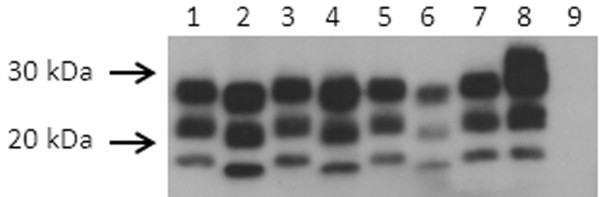
**Glycoform analysis of PrP**^**Sc **^**in Tg338 mice inoculated with different isolates of small ruminant scrapie.** Relative densities of unglycosylated, monoglycosylated and diglycosylated forms of PrP^Sc^ bands were obtained for each P2 mouse brain using an image analyzer. Green circle – inoculum group 3558; red circle – inoculum group 3538; black circle – inoculum group 30–75; blue circle – inoculum group 3178.

Glycoform analysis was performed on PK-digested homogenates of P2 Tg338 brain from each of the 4 inoculum groups (1 reference sheep and 3 goats with naturally occurring scrapie). Western blots were scanned and the percent of the total density represented in each of the 3 PrP^Sc^ bands was plotted. Although identical wet weight equivalents (300 μg) of each P2 mouse brain homogenate were loaded, the relative amount of PrP^Sc^ in each sample slightly varied among mice (Additional file 1). The relative amounts of the PrP^Sc^ bands were diglycosylated > monoglycosylated > unglycosylated (Figure 5 and Additional file 2). Notwithstanding minor differences in relative band densities, graphic display on a tri-plot clearly shows clustering of all 4 four scrapie isolates (Figure 5).

### Histologic scoring of vacuolization by brain region of Tg338 mice

Distribution and severity of vacuolization was assessed in P2 Tg338 mice from each of the 4 inoculum groups. Twelve regions (9 grey matter, 3 white matter) [15] of each second passage Tg338 mouse and non-inoculated, age-matched mice were scored for vacuolization by a pathologist (TRS) without knowledge of inoculum group. Vacuolization was not observed (score = 0) within any of the twelve brain regions of non-inoculated mice except for within G2 where a score of “1” was recorded for one mouse; the mean (± standard error) score for region G2 in non-inoculated mice was thus 0.125 ± 0.354 (n = 8 mice), which is not significantly different than a score of 0.

**Figure 6:**
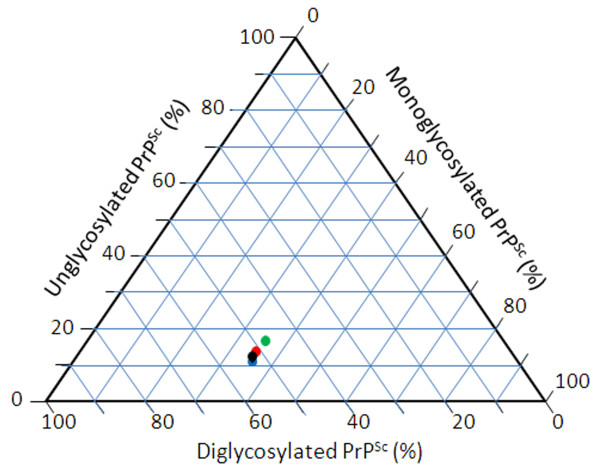
**Histological vacuole scores by brain region for small ruminant scrapie in P2 Tg338 mice.** Shown are the mean vacuolization scores with standard errors by Tg338 mouse brain region for scrapie isolates derived from the brain of three clinical goats and a reference sheep. Each isolate source case number is indicated to the right in each panel. Note the similar regional pattern of grey (G) and white (W) matter vacuole scores induced by goat and sheep-derived scrapie. G1, dorsal medulla nuclei; G2, cerebellar cortex of the folia including the granular layer adjacent to the fourth ventricle; G3, cortex of the superior colliculus; G4, hypothalamus; G5, thalamus; G6, hippocampus; G7, septal nuclei of the paraterminal body; G8, cerebral cortex (at the level of G4 and G5); G9, cerebral cortex (at the level of G7); W1, cerebellar white matter, W2, tegmentum; and W3, pyramidal tract.

Mean vacuolization scores and variation for P2 Tg338 mice are shown in Figure 6. The mean vacuolization scores in all inoculum groups were significantly greater than 0 in brain regions G1, G4, G5, G7, and W2 and W3, but were not significantly different from zero in regions G2, G6, G8, G9 and W1. Mean vacuolization scores in region G3 were the most variable: the mean vacuolization score was significantly increased for groups derived from sheep 3178 (n = 10) and goat 3538 (n = 11) but not for goats 3558 (n = 4) and 30–75 (n = 5).

### PET blots

**Figure 7:**
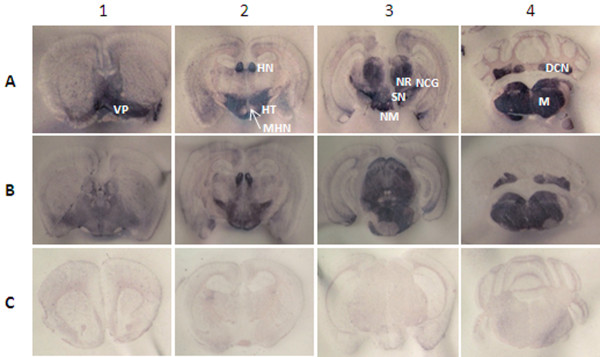
**Distribution of PK-resistant PrP labeling of PET blots.** Shown is the neuroanatomic distribution of PrP^Sc^ at 4 different brain levels of P2 Tg338 mice representing inoculum groups 30–75 **(A)** and 3178 **(B)**, and an age-matched uninoculated control group mouse **(C)**. Brain sections were dissected at the levels of frontal cortex (level 1), hypothalamus (level 2), hippocampus (level 3) and cerebellum-medulla (level 4). Blots were probed with mAb F99/97.6.1. VP, ventral pallidum; HT, hypothalamus; MHN, medial hypothalamic nucleus; HN, medial habenular nucleus; NM, nucleus mammillaris; NR, nucleus rubor; SN, substantia nigra; NCG, nucleus corporis geniculati; DCN, deep cerebellar nuclei; M, medulla.

Paraffin embedded tissue (PET) blot analysis was performed on brain sections from one mouse in each P1 inoculum group and 4 to 9 mice in each of the P2 groups. Representative data are shown in Figure 7. No labeling was detected in brains from negative controls (Figure 7C) or in samples labeled with an isotype control antibody (data not shown). PK-resistant PrP was visible at all 4 anatomic levels in sections from infected mice, with dense labeling in the striatum, ventral pallidum, claustrum, insular cortex, paraterminal body and cingulated cortex at level 1, the thalamus and hypothalamus at level 2, the superior colliculus and midbrain at level 3 and deep cerebellar nuclei and medulla at level 4. There was no evidence of species-specific discriminatory labeling and no significant variation within the 3 caprine scrapie groups.

## Discussion

Bioassay of tissues from scrapie infected sheep and goats demonstrated the transmissible nature of the disorder and the novel characteristics of the infectious agent well before the introduction of the prion hypothesis [16,17]. Oral and parenteral challenge demonstrated the susceptibility of goats to ovine scrapie [18-20]. Reciprocal studies examining the susceptibility of sheep to caprine scrapie are less well described. Studies in the natural ruminant host are limited by the prolonged incubation time and the expense of housing large animals in biocontainment. Transgenic mice expressing a PRNP gene from a species of interest are a suitable surrogate host for some studies of prion disease in humans, cattle, sheep and deer [15,21-27]. Considerable variation is noted among transgenic strains, however, with differences in outcome associated with route of inoculation [28,29], transgene sequence of the regulatory and coding regions of PRNP [30], and transgene expression level [31]. The murine Tg338 line expresses the ovine PRNP VRQ allele under the ovine PRNP promotor with overexpression of PrP^c^ in the central nervous system and lymphoreticular system [29], sites of PrP^Sc^ accumulation in sheep and goats. This transgenic mouse line has been useful in characterizing atypical (Nor98) [32,33] and classical scrapie in sheep of various genotypes [34,35]. Several ovine isolates have been further characterized by serial passage to identify candidate classical scrapie strains differing in biochemical or clinical profiles in Tg338 mice [36,37]. In this study, we examined the use of Tg338 mice as a surrogate for characterizing caprine scrapie bioassayed in the ovine host. We examined incubation time, neuropathological phenotype, glycoform profile, and proteinase K cleavage pattern following primary and secondary passage of brain homogenates from three goats with naturally acquired scrapie. Brain tissue from a sheep homozygous for the PRNP ARQ allele was used as a reference ovine scrapie case.

Incubation times for all 4 inoculum groups were prolonged and variable upon primary passage to Tg338 mice; mean incubation times were not related to ELISA/Western blot titer. The sample from goat 3538 showed the widest variation in survival time on primary passage, with insidious onset at as early as 199 days and clinical signs of ataxia, weight loss and kyphosis by 206 days; survival times in some mice in this group exceeded 300 days. Mice were euthanized at 199–228 days (n = 3 short incubation), at 253–263 days (n = 4 mid-range incubation), and at greater than 331 days (n = 3 long incubation). Subpassage of samples from mice in the short (228 days) and long (331 days) incubation groups resulted in shorter incubation periods and mean survival times with no significant difference between the short and long incubation sample recipients (137 +/−2 and 135 +/−5 respectively); likewise, there was no significant difference among the groups of P2 mice from the other inocula.

Several phenotypes have been described following passage of tissue from scrapie infected sheep to Tg338 mice. Notably, differences in incubation time on primary and secondary passage associated with differences in electrophoretic profiles have been described [36,38], particularly in sheep with alleles occurring on the ARQ haplotype. These cases have been reported as homozygous for alleles encoding ARQ at codons 136, 154 and 171, and the effect of other polymorphisms (nonsynonymous changes at codon 141 or 241 for instance, on the ARQ backbone in donor sheep) has not been determined. Multiple electrophoretic patterns arising from a single ovine homogenate occur as individual or mixed profiles, even when this variation was not noted in the original ovine sample [38]. In our study, the electrophoretic profiles from all murine samples showed only a fast migrating unglycosylated band corresponding to a reduction of ~1.5 to 2 kDa in apparent molecular mass when compared to the ruminant derived samples. The shift in PK cleavage site was confirmed by marked reduction of reactivity by mAb P4, which recognizes an epitope at ovine residues 93–99. Western blot analysis with antibody SAF84 showed no evidence of a 14 kDa band, ruling out a strain CH1641 pattern [14]. The pattern of prolonged incubation associated with reduced apparent molecular weight product in Western blot is consistent with ARQ isolates characterized by Thackray et al. [34,36], Beck et al. [38], and Tixador et al. [39]. Our observation was not based on an inherent characteristic of the Tg338 mouse line or our methods, as samples from mice infected with VRQ ovine tissues migrate at ~21 kDa (data not shown).

PET blots, glycoform analysis, and lesion profiles of the Tg338 samples derived from caprine brain inoculation were remarkably consistent. The marked uniformity in survival time following secondary passage and the consistent patterns in PET blot, glycoform analysis and electrophoretic profile suggest that the caprine isolates, derived from separate herds, represent a single strain with characteristics of a predominant US ovine scrapie strain. This finding is not surprising; scrapie appears to have been introduced into the US in sheep and is observed almost exclusively in ARQ homozygous sheep. Subsequent spread to goats is reported only rarely and there may be a limited number of caprine scrapie strains in the population. The US will expand caprine scrapie surveillance over the next 3 years as ovine scrapie nears eradication; as additional samples from goats are identified, monitoring of caprine scrapie strains in the US using biochemical methods and bioassay in Tg338 mice should be useful in identifying any additional strains.

## Conclusions

The Tg338 mouse strain expressing the VRQ allele of the ovine PRNP gene is useful in detection and analysis of ovine prions. In this study, this murine strain was found to propagate prions following intracerebral inoculation with brain homogenates from goats with naturally occurring scrapie. The electrophoretic profile and incubation time on secondary passage were similar to those observed by passage of ARQ homozygous sheep. The high attack rate and uniform findings on PET blot, glycoform analysis and lesion profile provide a suitable baseline for examination of caprine samples identified in the expanded US caprine scrapie surveillance program.

## Methods

### Animals and PRNP genotyping

The Washington State University and University of Washington Institutional Animal Care and Use Committees granted approval for the study before it was conducted, under agreements 3811, 3815 and 1640. Breeding pairs of transgenic Tg338 mice were kindly provided by Hubert Laude and held in a breeding colony at the University of Washington. The presence of the transgene was confirmed by DNA analysis of tail snips.

Scrapie infected goats and sheep were diagnosed by antemortem analysis of rectal mucosal associated lymphoid tissue [40] or post mortem analysis of brain and retropharyngeal lymph node for evidence of PrP^Sc^ deposits detected by monoclonal antibody immunohistochemistry [41]. Donor tissue was collected from three domestic mixed breed goats (case numbers 3558, 3538, and 30–75) and one reference sheep (case number 3178). The animals were acquired from separate flocks, although 30-75 and 3538 were born at the same facility. Postmortem confirmation was performed at the National Veterinary Services Laboratory, USDA, Ames IA, USA, using immunohistochemistry analysis of brain using monoclonal antibody (mAb) F99/97.6.1.

Diploid PRNP genotypes were determined by polymerase chain reaction amplification of the open reading frame followed by DNA sequence analysis of both strands using standard methods [12,42].

### Mouse inoculations

Brain tissue from the three goats with naturally acquired scrapie and the reference sheep with classical ovine scrapie were prepared as 10% (w/v) homogenates in normal saline. Relative titer of each homogenate was estimated from serial dilution and endpoint titration using a commercial enzyme linked immunosorbent assay and western blot analysis as described below. Total protein levels varied slightly among the inocula: 2 mg starting wet weight tissue yielded 76 (goat 3538), 71 (goat 3558), 85 (goat 30–75) and 99 (sheep 3178) μg total protein as determined by BCA protein assay. Passage 1 (P1) Tg338 mice were inoculated with 20 μl of 10% (w/v) sheep or goat brain homogenate (2 mg starting wet weight tissue) by the intracerebral route in the right parietal lobe. Serial passage of murine brain (passage 2, P2) was performed using 20 μl of a 10% (w/v) homogenate of brain from clinically affected P1 mice administered by the intracerebral route as above. An additional group of mice was inoculated intraperitoneally with brain homogenate from sheep 3178 using 2 mg starting wet weight tissue in a final volume of 200 μl. Inoculated animals and age matched uninoculated controls were monitored daily for appearance of clinical signs suggestive of scrapie (weight loss, lethargy and kyphosis) and culled at terminal disease. Brain and spleen were collected from mice at necropsy. Samples were fixed in 10% buffered formalin or frozen at–20C for western blot analysis.

### Incubation time

Survival time following intracerebral challenge with brain homogenates from goats or sheep (P1) or mouse (P2) was recorded as days post-inoculation (dpi). Recipient animals terminated for reasons other than clinical scrapie disease were considered censored data. The survival function of each scrapie isolate was determined using the Kaplan-Meier product-limit estimation method (LIFETEST procedure, SAS for Windows version 9.2; SAS Institute Inc., Cary, NC, USA). Median survival times with 95% confidence limits were determined by the log(−log) method.

### Relative PrP^Sc^ levels in goat and sheep inoculum

Relative amounts of PrP^Sc^ in brain homogenates from three goats and one reference sheep with scrapie were determined by serial endpoint dilution followed by enzyme linked immunosorbent assay and Western blot analysis of paired samples. Aliquots of the 10% homogenate of sheep or goat tissue prepared for inoculation were adjusted with 2 volumes of lysis buffer (0.5% deoxycholic acid, 0.5% NP-40, 10 mM Tris–HCl, pH 7.5) using a table top homogenizer (Fastprep, Thermo Electron Co., Waltham, MA, USA). Preliminary assays demonstrated that the use of this lysis buffer did not affect ELISA readings (data not shown) and the initial dilution in lysis buffer provided a homogeneous mixture suitable for aliquoting into paired samples for ELISA and Western blot. Additional serial two fold dilutions were prepared in lysis buffer and assayed in duplicate using a commercially available PrP^Sc^ detection kit (ELISA) (HerdCheck Scrapie-BSE Antigen Test, IDEXX Laboratories, Westbrook, ME, USA). Mean absorbance values (A_450_) were plotted against starting wet weight equivalents of brain. The cutoff value was determined using the internal standard provided in the kit.

### Western blot analysis

PrP^Sc^ was detected by Western blot assay as described previously [41-43] with minor modifications. Aliquots of the 10% (w/v) brain homogenates from sheep, goats, or mice serially diluted in lysis buffer for ELISA (above) were assayed by Western blot. Homogenates were incubated with proteinase K (PK) at 100 μg/mL final concentration at 37C for 90 min for murine homogenates and 50 μg/mL final concentration at 37C for 60 min for ovine and caprine homogenates. For the endpoint titration of sheep and goat inocula, the starting 10% homogenate in PBS was adjusted with two volumes of lysis buffer and treated with proteinase K (PK) at 50 μg/mL final concentration at 37C for 60 min. Serial two fold dilutions were prepared in lysis buffer, combined with sample loading buffer (NuPage, Invitrogen, Carlsbad, CA, USA), and loaded onto 12-well 12% Nu-PAGE Bis-Tris gels (Invitrogen). Starting wet weight equivalents for titration were 750 μg for ovine and caprine tissues. Murine tissues for glycoform analysis were assayed at 300 μg per lane. After electrophoresis, proteins were transferred onto polyvinylidene fluoride (PVDF) membranes (Millipore, Billerica, MA, USA), blocked with commercial casein blocker (Pierce, Rockford, IL, USA) and incubated with primary mAb F99/97.6.1 (3.5 μg/mL; O’Rourke et al., 2000), mAb P4 (0.2 μg/ml, R-BioPharm AG, Marshall, MI, USA) or mAb SAF84 (0.2 μg/ml, Cayman Chemical, Ann Arbor, MI, USA) followed by incubation with a peroxidase labeled goat anti-mouse IgG_1_ secondary Ab (Southern Biotech, Birmingham, AL, USA; 1:5,000, for detection of mAb F99/97.6.1) or a peroxidase labeled F(ab’)2 fraction of goat anti-mouse IgG_2_ (KPL, Gaithersburg, MD, USA, 1:20,000 for detection of mAb P4 and SAF84. Bound antibody was detected by chemiluminescence (Amersham ECL, GE healthcare, Piscataway, NJ, USA). Membranes were exposed to radiographic films (Kodak BioMax Chemiluminescence Films, Rochester, NY) for exposure times of 1–10 min. Digital images of the radiographic films were obtained with an Alpha Innotec image analyzer (Alpha Innotech Corp, San Leandro, CA, USA) and saved in tiff file format. Density of the diglycosylated band was plotted against starting wet weight of tissue to estimate relative endpoints.

### Glycoform analysis

Brain tissues of 20 inoculated mice (five from each donor group) were selected for glycoform analysis and tested in three independent western blot assays. For each sample, triplicate aliquots of 50 μl brain homogenate were incubated with PK (100 μg/mL final concentration) at 37C for 90 min. Aliquots equivalent to 300 μg wet brain weight equivalents were loaded into individual lanes of 12-well 12% polyacrylamide gels and analyzed by western blot assay as described above, using mAb F99/97.6.1. Digital images of the radiographic films were obtained with an Alpha Innotec image analyzer and saved in tiff file format. These files were analyzed with AlphaEaseFC software Version 4.0 to identify the relative densities of unglycosylated, monoglycosylated and diglycosylated forms of PrP^Sc^. For each group of mice, mean density of each band was plotted into Tri-plot Excel template. The tri-plot excel template was downloaded from http://www.lboro.ac.uk/research/phys-geog/tri-plot[44].

### Histopathology

Formalin fixed brains were processed routinely and sectioned at 5 μm for hematoxylin and eosin staining. Tissues were analyzed for vacuolation profile using standard methods as described [15]. Lesion severity was quantitated as described [45]. Briefly, scores were assigned on a scale of 0 to 5 for specific neuroanatomic areas based on the intensity of the vacuolation. Regions scored were G1, dorsal medulla nuclei; G2, cerebellar cortex of the folia including the granular layer, adjacent to the fourth ventricle; G3, cortex of the superior colliculus; G4, hypothalamus; G5, thalamus; G6, hippocampus; G7, septal nuclei of the paraterminal body; G8, cerebral cortex (at the level of G4 and G5); and G9, cerebral cortex (at the level of G7). In addition, three white matter areas were scored; W1, cerebellar white matter, W2, tegmentum, and W3, pyramidal tract. Regions were assigned 0 if vacuolation was similar to that observed in negative control mice; 1 for regions with a few unevenly scattered vacuoles, 2 for regions with a few evenly scattered vacuoles, 3 for regions with a moderate number of evenly scattered vacuoles, 4 for regions with many vacuoles, including some confluence, and 5 for regions with dense vacuolation with most of the field confluent. This evaluation was done by examination of the entire region on 20X.

### PET blots

Regional distribution and intensity of PrP^Sc^ labeling in P2 Tg338 mouse brains were evaluated in paraffin embedded tissue blots. The brain sections were dissected at the levels of frontal cortex (level 1), hypothalamus (level 2), hippocampus (level 3) and cerebellum-medulla (level 4) and probed with mAb F99/97.6.1 as described [46-48], with minor modifications [49]. Eight P2 scrapie inoculated Tg338 mice, including 2 from each inoculum group and 2 age-matched uninoculated negative control mice, were evaluated as described [48].

## Authors’ contributions

KIO developed the study design, contributed to design of the glycoform analysis, participated in histologic scoring, performed analysis of genotypes, and drafted the manuscript. DAS performed the survival curve analysis and the vacuole pattern analysis. TRS performed the histopathological examinations. RPD performed the glycoform analysis and assisted with PET blot analysis. DZ performed the western blots, ELISA and PET blots and provided data analysis for ELISA and Western blots. MAH analyzed the PET blot data. TCT performed the histologic preparation of all small ruminant and murine tissues. All authors participated in writing the manuscript and have read and approved the final manuscript.

## Supplementary Material

Additional file 1 ** Brain tissue from 20 Tg338 passage 2 mice (five from each sheep- or goat-inoculated P1 donor) were selected for western blot assay and glycoform analysis.** Three hundred μg wet brain weight equivalent volumes of each of the mouse brain were digested with proteinase K and loaded into SDS-PAGE gels followed by western blot assay using mAb F99/97.6.1 and chemiluminescent imaging. Films (representative film shown in Additional File 1) were used to obtain relative densities of un-, mono- and di-glycosylated forms of PrP^Sc^ bands for glycoform analysis. Click here for file

Additional file 2 ** Glycoform patterns of PrP**^**Sc **^** from 3 goats and a reference sheep with naturally occurring scrapie and from Tg338 mice from each inoculum group.** Click here for file
