# Supplementary figures and images for: Transmissibility of caprine scrapie in ovine transgenic mice
Source: BMC Vet Res. 2012 Apr 2;8:42. doi: 10.1186/1746-6148-8-42 (PMC3489715; doi:10.1186/1746-6148-8-42)

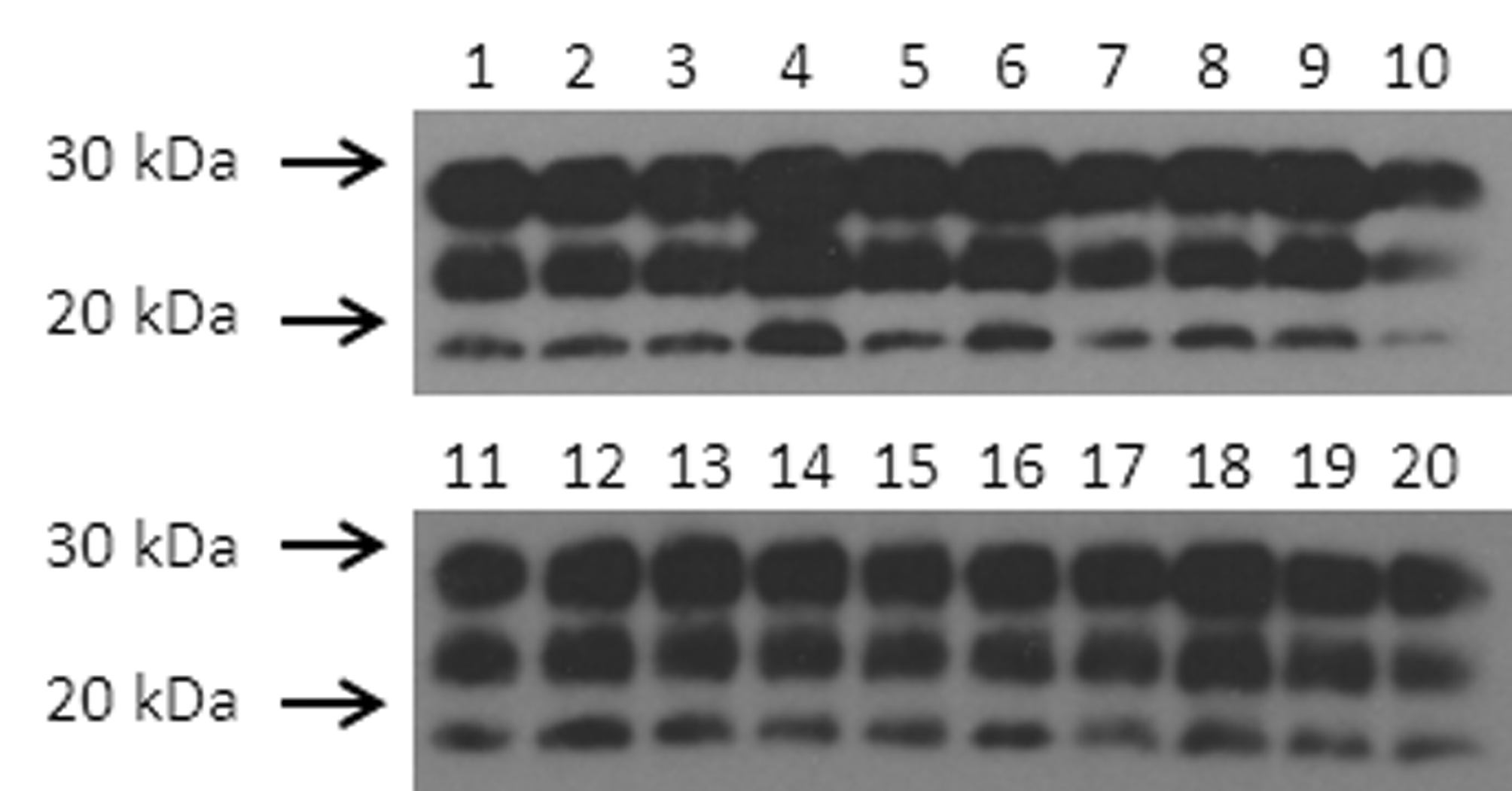

Supplement: Additional file 1 — Brain tissue from 20 Tg338 passage 2 mice (five from each sheep- or goat-inoculated P1 donor) were selected for western blot assay and glycoform analysis. Three hundred μg wet brain weight equivalent volumes of each of the mouse brain were digested with proteinase K and loaded into SDS-PAGE gels followed by western blot assay using mAb F99/97.6.1 and chemiluminescent imaging. Films (representative film shown in Additional File 1) were used to obtain relative densities of un-, mono- and di-glycosylated forms of PrPSc bands for glycoform analysis. [file 1746-6148-8-42-S1.tiff]
