# Supplementary material for: Transmissibility of caprine scrapie in ovine transgenic mice
Source: BMC Vet Res. 2012 Apr 2;8:42. doi: 10.1186/1746-6148-8-42 (PMC3489715; doi:10.1186/1746-6148-8-42)
Supplement: Additional file 2 — Glycoform patterns of PrPSc from 3 goats and a reference sheep with naturally occurring scrapie and from Tg338 mice from each inoculum group. [file 1746-6148-8-42-S2.docx]

**Table 2: Glycoform patterns of PrP^Sc^ from 3 goats and a reference sheep with naturally occurring scrapie and from Tg338 mice from each inoculum group**

| Sample^1^ | Diglycosylated band^2^ | Monoglycosylated band^2^ | Unglycosylated band^2^ |
| --- | --- | --- | --- |
| Sheep 3178 | 57.9 | 33.4 | 8.7 |
| Tg338 P1 3178 | 59.73 (2.5) | 31.50 (0.79) | 8.77(1.85) |
| Tg338 P2 3178 | 53.92 (6.54) | 34.94 (3.27) | 11.14 (4.95) |
| 30-75 | 51.7 | 32.3 | 16.1 |
| Tg338 P1 30-75 | 55.07 (1.7) | 31.90 (1.85) | 13.03 (1.75) |
| Tg338 P2 30-75 | 53.36 (10.02) | 34.65 (7.69) | 11.94 (3.34) |
| 3538 | 51.35 (5.39) | 29.53 (3.21) | 14.73 (3.34) |
| Tg338 P1 3538 | 55.4 (6.08) | 32.4 (2.55) | 12.25 (3.46) |
| Tg338 P2 3538 | 52.26 (4.34) | 34.34 (2.66) | 13.39 (2.66) |
| 3558 | 56.75 (4.6) | 32.95 (0.05) | 10.2 (2.4) |
| Tg338 P1 3558 | 54.7 | 33.5 | 11.8 |
| Tg338 P2 3558 | 49.23 (2.51) | 34.55 (1.29) | 16.20 (2.5) |

^1^Brain homogenates from small ruminants (1 sheep and 3 goats) with naturally acquired scrapie and first (P1) or second (P2) passage Tg338 mice inoculated with the small ruminant brain homogenates

^2^Percent of the total density in scanned western blots in each of the three major proteinase K resistant bands
